# Effects of *Cleistanthus collinus* on the reproductive system of male Wistar rats

**DOI:** 10.5935/1518-0557.20210114

**Published:** 2022

**Authors:** Subramanian Umamaheswari, Chandrashekaran Girish, Debdatta Basu

**Affiliations:** 1 Department of Pharmacology, Jawaharlal Institute of Postgraduate Medical Education and Research, Puducherry, India; 2 Department of Pathology, Jawaharlal Institute of Postgraduate Medical Education and Research, Puducherry, India

**Keywords:** apoptosis, *Cleistanthus collinus*, cyclophosphamide, hormones, oxidative stress, spermatogenesis, testis

## Abstract

**Objective:**

Plants are widely used in the traditional system of medicine and many of them can adversely affect the reproductive system. *Cleistanthus collinus* is a plant containing many active phytochemicals, which have the potential to be developed as a drug. The present study was aimed to evaluate the effects of an aqueous extract of *C. collinus* on the male reproductive system.

**Methods:**

Male Wistar rats were treated with different doses of *C. collinus* (200 and 400 mg/kg, orally), or with saline for 28 days and its effect on the reproductive system was assessed. Cyclophosphamide (100 mg/kg, weekly, i.p), a well-known reproductive toxicant, was used as a positive control.

**Results:**

There was a significant reduction in sperm count and motility with *C. collinus* treatment, along with the destruction of primary spermatocytes, spermatids and reduced thickness of the basal layer. The LH, FSH and testosterone levels were reduced significantly. A reduction in the area of seminiferous tubules indicates the destruction of germ cells and Sertoli cells. *C. collinus* treatment increased the oxidative stress, evidenced by elevated malondialdehyde levels along with a reduction in catalase and GSH activities. The expression of BAX, BCL-2 and p53 was observed in spermatocytes, indicating an increase in apoptosis.

**Conclusions:**

*C. collinus* can produce male reproductive toxicity, which may be mediated through alterations in hormonal levels, which in turn interferes with spermatogenesis. It may increase the expression of apoptotic factors and deplete protective antioxidant enzymes in the testis.

## INTRODUCTION

Plants help mankind in various ways, providing food, medicine, oxygen and maintaining the environment (Fridlender *et al.*, 2015). Plant products are known for both therapeutic and toxicological profile (George, 2011). Medicinal plants can be used as contraceptives, emmenagogues and abortifacients (D'Cruz *et al*., 2010). Some of them are reported to enhance reproductive processes; but, on the other hand, they can also hinder testicular functions. Many plants used therapeutically, can adversely affect the male reproductive system. Neem and *Carica papaya* extract are reported to impede various stages of testicular spermatogenesis in animal models. The reduction in the levels of testosterone, luteinizing hormone (LH) and follicle stimulating hormone (FSH) was reported with garlic and *Quassia amara* extracts in male albino rats (D'Cruz *et al.*, 2010). The oral administration of *Azadirachta indica* leaves has produced regression and a decrease in the number of Leydig cells, and their nuclear diameter, indicating androgen deficiency (D'Cruz *et al.*, 2010). Therefore, knowledge of the toxic profile of the plants on reproduction is important before using them therapeutically.

*Cleistanthus collinus* is a plant species that belongs to the Euphorbiaceae family, which vernacular name in Tamil is oduvanthalai. In Hindi it is called Garari; Telugu, as Vadise; Malayalam, as Nilapala; and Bengali, as Karlajuri (Eswarappa *et al.*, 2003). It is widely distributed in South Asia. Leaves of this plant are consumed for suicidal purposes, and to induce abortion. Mainly decoction of leaves from this plant is used for suicide. The patient presents with abdominal pain, dyspnea, chest pain, giddiness and headache. Signs of poisoning include hypotension, ST depression, acid-base imbalance and electrolyte imbalance (Benjamin *et al.*, 2006). The mortality rate due to *Cleistanthus collinus* poisoning is around 30%. Hypotension caused by *Cleistanthus collinus* poisoning paved the way for the identification of vasodilatory property of *Cleistanthus collinus* (Benjamin *et al.*, 2006; Parasuraman *et al.*, 2009). Later individual active components of this plant were isolated and studied. The most notable compounds are cleistanthin A, cleistanthin B, diphyllin and collinusin (Parasuraman *et al.*, 2009). Though it is a poisonous plant, the active components of this plant have been explored for therapeutic properties such as antihypertensive, diuretic and anticancer agents (Parasuraman *et al.*, 2011; Pradheepkumar *et al.*, 2000; Parasuraman & Raveendran, 2012).

Promising active components of *Cleistanthus collinus* will have a fair chance to emerge as a drug. Toxicity studies in animals help identify the adverse effects of the new compounds, which are to be tested in humans. A sub-chronic toxicity study on *Cleistanthus collinus* was done by Parasuraman *et al.* (2014). Their study focused on the effect of *Cleistanthus collinus* on the cardiovascular system, respiratory system and gastrointestinal system (Parasuraman *et al*., 2014). Though an increase in the testis weight was reported, no study evaluated the effects of *Cleistanthus collinus* on the male reproductive system in morphological, hormonal and histopathological aspects. Hence the present study was aimed to evaluate the effect of an aqueous extract of *Cleistanthus collinus* exposure on the male reproductive system in rats.

## MATERIALS AND METHODS

### Chemicals and Drugs

All the chemicals were of analytical grade. Trichloroacetic acid (Sigma, India), 2-Thiobarbituric acid (Sigma, India), Sulfosalicylic acid (Avra, India), Dithiobis-2 nitro benzoic acid (DTNB) (Himedia, India), EDTA (Ethylenediaminetetraacetic acid) disodium (REACHEM, India), Sodium chloride (REACHEM, India), Sodium dihydrogen phosphate (Excelar, India), Sodium hydrogen monophosphate (Excelar, India), Hydrogen peroxide-(CHEMSURE, India), Reduced glutathione (Himedia, India), Cyclophosphamide (Endoxan N 500 mg) were procured from commercial suppliers.

### Plant extract preparation

Leaves from *Cleistanthus collinus* were obtained from the village near Sedharapet, Puducherry, India, in the month of June 2016. Then they were confirmed and certified by a botanist. The leaves were dried under shade for four weeks and after drying these leaves were ground into powder form with the help of a mixer. Then the powdered leaves were weighed, 100 grams of leaf powder were dissolved in 1000 mL of distilled water. The dissolved *Cleistanthus collinus* leaf powder was boiled at 100˚C. After 1h of boiling, the decoction was cooled under running tap water and was filtered with nylon mesh. The decoction was then condensed and the resultant aqueous extract of *Cleistanthus collinus* was in the form of crystals, which was stored in the refrigerator. The required quantity of crystal forms of the aqueous extract of *Cleistanthus collinus* was dissolved in normal saline at the time of administration. *Cleistanthus collinus* leaf powder of 1.5 kg was used and the extract obtained was 111g. The yield of this plant was 7.4%.

Dose selection: The lethal dose 50% (LD 50) of *Cleistanthus collinus* is 8g/kg. Hence 10% of it, which was 800 mg/kg, was considered as a toxic dose (Jayanthi *et al.*, 2009; Almança *et al.*, 2011). Two lower graded doses of 400 and 200 mg/kg were selected for the study.

### Animals

Animal care and experiments complied with the CPCSEA guidelines, The Government of India. The study was approved by the Post-graduate Research Monitoring Committee and the Institute of Animal Ethics Committee, JIPMER, Puducherry. Healthy male Wistar rats (24 animals) of 2-3 months old, weighing 200-250g were obtained from the Central Animal House, JIPMER, Puducherry. The procured animals were kept in the animal house of the Department of Pharmacology, in plastic transparent cages. The animals were acclimatized for 7 days. Three animals were grouped in a single cage. Continuous access to rat chow and drinking water were provided, and a 12 h light/12 h dark cycle was maintained. The temperature was set at 22˚C and humidity was maintained at 70%.

### Experimental design

Adult male Wistar rats (n=24) were divided randomly into five groups, 6 animals per group. All the animals were weighed before the start of the experiment.

Group 1, control group: the animals received 2 mL of normal saline once daily for 28 days through oral gavage.

Group 2, positive controls: the animals received cyclophosphamide at the dose of 100 mg/kg through the intraperitoneal route, once weekly for 4 weeks.

Group 3, the animals received a 200 mg/kg dose of *Cleistanthus collinus* for 28 days through oral gavage.

Group 4, the animals received a 400 mg/kg dose of *Cleistanthus collinus* for 28 days through oral gavage.

### Blood and tissue collection

The animals were anesthetized with isoflurane inhalation and the blood sample was collected by cardiac puncture. The coagulated blood was centrifuged under 3500 rpm for 10 min, and the serum was separated for hormonal assays (LH, FSH and testosterone). After the blood collection, the animals were sacrificed by cervical dislocation and laparotomy was conducted. Both the testes and epididymis were removed. The right testis was processed for histopathological and immunohistochemical examinations. The left testis was homogenized with the help of a homogenizer in phosphate-buffered saline and centrifuged at 3500 rpm for five min, and the supernatant was further centrifuged at 10,000 rpm for 45 min. The supernatant was stored in -80˚C for antioxidant assays (GSH-reduced glutathione, catalase) and a malondialdehyde (MDA) assay.

### Analysis of sperm parameters

Body weight, testis and epididymal weight:

The body weight of the rats was measured at the beginning of the study and at weekly intervals. At the end of the study, i.e on the 29^th^ day, the animals were slaughtered, the testes and epididymis were removed and weighed using an electronic weighing balance.

Sperm motility and count: The cauda epididymis was dissected and minced in 1 ml of phosphate-buffered saline and the dilution was made in the ratio of 1:40. Then the sperm parameters were assessed under a light microscope (10X). The sperm count was determined using a hemocytometer. The sperm was counted in all hemocytometer chambers, except for the central red blood corpuscles (RBC) chamber. The sperm were counted from left to right. After counting the total sperm in each chamber, their motility was also assessed and we calculated the percentage of motile sperms (Tripathi & Jena, 2008; Narayana *et al.*, 2012).

Sperm morphology assay: The fine epididymal sperm suspension was made and stained with 0.2 mL of 1% aqueous eosin. About one drop of stained suspension was placed on a clean slide and dried. The slides were examined under the microscope (40X), for abnormalities in 500 sperms per animal, and were classified into normal and abnormal sperms. The abnormal sperms were classified under head abnormalities and tail abnormalities, then we calculated the percentage of sperm abnormalities (Narayana, 2008; Johnsen, 1970).

### Macroscopic and microscopic examination of testis

The right testes were fixed in formaldehyde solution for 24h and was processed for paraffin embedding. The sections were analyzed for any histopathological changes, including the presence or absence of vacuoles, gaps and abnormal cells.

Johnsen's scoring and area of the seminiferous tubule: Johnsen's testicular scoring was performed for the control and study groups. All cross-sectioned tubules were evaluated systematically, and a score between 1 (very poor) and 10 (excellent) was given to each tubule according to Johnsen's criteria (Johnsen, 1970). Twenty-five tubules were evaluated for each animal.

Area of seminiferous tubules: we assessed each testis using a calibrated ocular micrometer. We randomly measured 30 rounded section of seminiferous tubules (Narayana, 2008).

### Immunohistochemistry of testis for p53, BCL2 and BAX

Tissue sections were deparaffinized in xylene (2 X 5 min), hydrated in ethanol (2 X 3 min 100% ethanol, 1 X 1 min 95% and 1 X 1 min 80% ethanol), and then rinsed in distilled water. Antigen retrieval was done by the heat retrieval method, where the slide rack immersed in Tris-buffer (pH - 9.9) in a pressure cooker. The sections were rinsed in PBS-Tween 20 and incubated with protein block for 10 min at room temperature. Then the sections were incubated with primary antibodies for p53 (pre-diluted, Bio SB, USA, Cat no: BSB 5841) BCL-2 (pre-diluted, Bio SB, USA, Cat no: BSB 6541) and BAX (pre-diluted, Bio SB, USA, Cat no: BSB 6078) for 1 h. at room temperature. The sections were rinsed in PBS-Tween 20 and treated with secondary antibodies (Bio SB, USA, Cat no: BSB0201), at a 1:100 dilution for 18 min at room temperature. After rinsing in PBS-Tween 20 the sections were covered with diaminobenzidine tetrahydrochloride (DAB) for 5 min at room temperature. The sections were counterstained with Mayer's hematoxylin and dehydrated in ascending grades of ethanol (95 % ethanol - 2 min, 100 % ethanol - 2x3 min). Finally, the sections were mounted in a DPX (Distyrene Plasticizer Xylene), and the coverslip was added.

### Hormonal assay

LH, FSH and testosterone levels in serum were measured by the ELISA method using commercial kits (Bioassay Technology Laboratory, Shanghai, China). The assays were performed according to the instructions, given in the catalogue (LH, Cat no: EA0013Ra, FSH, Cat no: EA0015Ra, and testosterone Cat no: E0259Ra).

### Oxidative stress parameters of testis

The supernatant of the tissue homogenate was used for oxidative stress parameter as follows:

Estimation of malondialdehyde (MDA): Malondialdehyde was assayed using thiobarbituric acid reacting substance (TBARS) by the method described by Utley *et al*. (1967). MDA was expressed in nanomoles/ml.

Estimation of reduced glutathione (GSH): Reduced glutathione, a non-enzymatic antioxidant was estimated by the method described by Smith *et al*. (1988). Dithiobis-2 nitro benzoic acid (DTNB) is a disulfide chromogen, which gets reduced by sulfhydryl compounds to produce a yellow-colored product. The absorbance of this colored chromogen is measured at 412 nm, which is directly proportional to GSH concentration.

Estimation of antioxidant enzyme, catalase: The activity of catalase was assayed by the method of Aebi (1984).

### Statistical analysis

Body weight was measured at baseline and after four weeks of treatment. The change in body weight and epididymal weight were analyzed by one way ANOVA, followed by the Tukey Krammer test (post hoc test). Testicular weight, sperm count, LH levels, Johnson's testicular scoring, area of seminiferous tubule and the levels of FSH, testosterone, catalase, MDA and GSH were analyzed by the Kruskal Wallis test, followed by Dunn's multiple comparison post-hoc test. A *p-*value of ≤0.05 was considered statistically significant.

## RESULTS

### Effect of *Cleistanthus collinus* on body weight, testicular and epididymal weight

The animals received saline (control group) or *Cleistanthus collinus* (200 and 400 mg/kg), through oral gavage daily for 28 days. Cyclophosphamide was given intraperitoneally once a week for four weeks.

There was a gain in body weight in the control group, whereas in the *Cleistanthus collinus* groups (200 and 400 mg/kg) and cyclophosphamide-treated group, there was a significant reduction in body weight (Table 1). There was a significant reduction in testicular weight in the cyclophosphamide and *Cleistanthus collinus* groups. Epididymal weight was reduced in the cyclophosphamide-treated group (*p*<0.05) and in the *Cleistanthus collinus* group - 400 mg/kg; though not statistically significant.

**Table 1.** Effect of *Cleistanthus collinus* on body weight, testicular weight and epididymal weight of Wistar rats.

	Control (saline)	Cyclophosphamide 100 mg/kg	*C. collinus* 200 mg/kg	*C. collinus* 400 mg/kg
Change in body weight (g)	52.16±13.22	-59.16±7.49^c^	-39±9.12^b^	-51.66±12.86^b^
Testes (g)	1.22 (1.01-1.25)	0.79 (0.72-0.89)^c^	0.95 (0.9-1.26)^b^	0.89 (0.86-0.98)^b^
Epididymis (g)	0.45±0.03	0.38±0.06^a^	0.44±0.04	0.39±0.03

(-) Indicates reduction in body weight. Rats received saline or plant extract through oral gavage daily for 28 days. Cyclophosphamide was given through the intraperitoneal route once a week for four weeks. Data are represented as mean ± SD or median (IQR), n=6.

a *p*<0.05,

b *p*<0.01,

c *p*<0.001 compared to the control group.

The parametric test used was one way ANOVA with the Tukey Krammer post hoc test and the Non-parametric test used was the Kruskal Wallis with Dunn’s multiple comparison post hoc test, based on the normality testing.

### Effect of *Cleistanthus collinus* on sperm parameters

**Figure 1:**
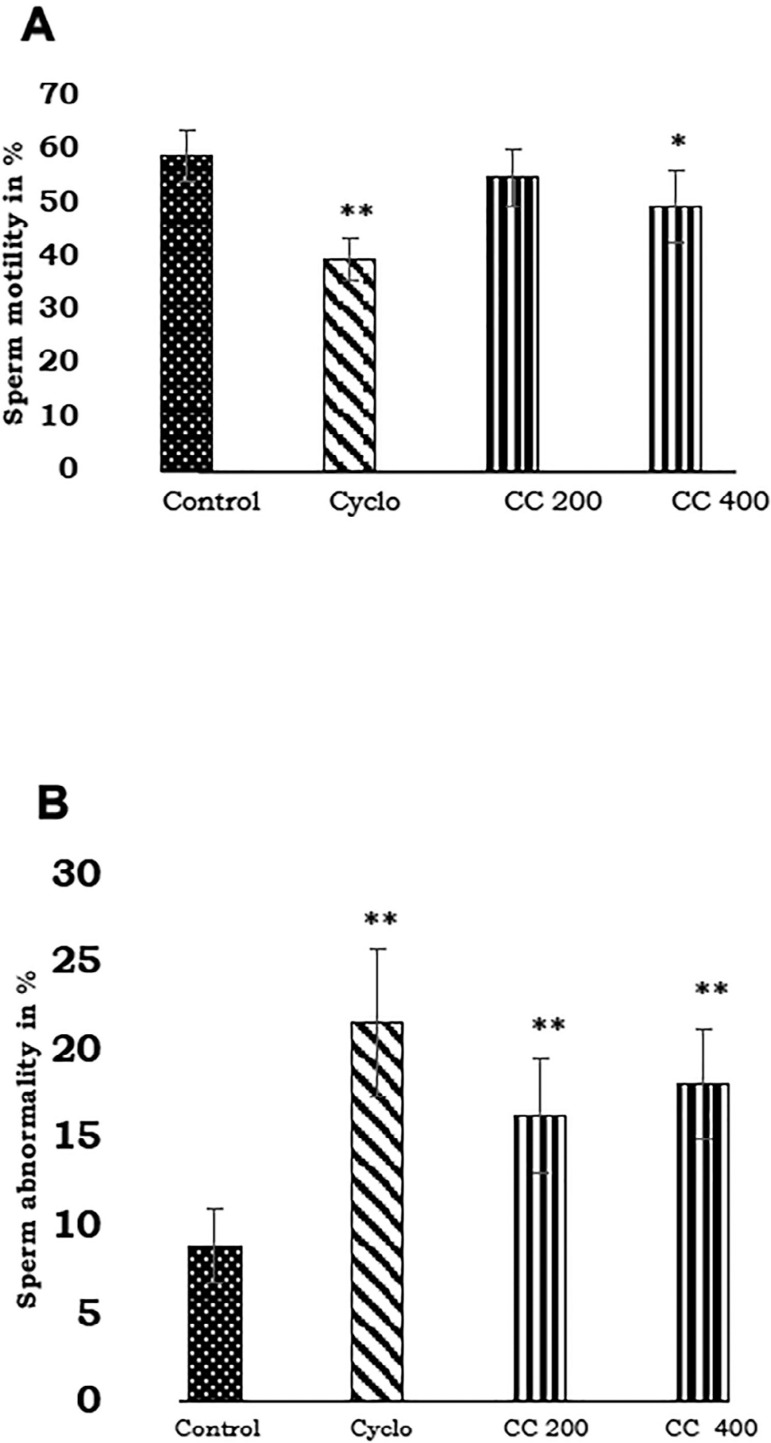
Effect of *Cleistanthus collinus* on sperm motility and sperm morphology of Wistar rats. Data are expressed as mean ± SD, n=6. *p< 0.05, **p<0.001 compared with control group. The statistical test used was One way ANOVA with Tukey Krammer post hoc test. Cyclo-cyclophosphamide, 100 mg/kg, CC 200-*Cleistanthus collinus*, 200 mg/kg CC 400- *Cleistanthus collinus*, 400 mg/kg.

There was a statistically significant reduction in sperm count and motility in the *Cleistanthus collinus* 400 mg/kg group (*p*<0.05), and in the cyclophosphamide-treated group (*p*<0.01) in comparison with the control group (Table 2 & Figure 1A). Morphological abnormalities in sperm were seen in the *Cleistanthus collinus* (200 and 400 mg/kg) groups and cyclophosphamide-treated groups (*p*<0.001, Figure 1B).

**Table 2.** Effect of *Cleistanthus collinus* on sperm count and testicular parameters of Wistar rats.

	Control (Saline)	Cyclophosphamide (100 mg/kg)	*C. collinus *(200 mg/kg)	*C. collinus* (400 mg/kg)
Sperm count (x 10^6^)	51.52 (38.35-54.65)	19.75 (18.0-42)^c^	35.42 (33.65-47.0)^d^	29.32 (28.65-47.5)^b^
Area of seminiferous tubules (mm^2^)	584.83±16.01	374.50±46.68^c^	385.83±67.88^c^	356.0±47.19^c^
Johnson’s scoring	9.40 (9-9.9)	7.00 (0-8.1)^b^	8.65 (8-9.4)	7.30 (5.2-8.1)^b^

Rats received saline or plant extract through oral gavage daily for 28 days. Cyclophosphamide was given through the intraperitoneal route once a week for four weeks. The following day, the animals were slaughtered, and the parameters were assessed. The data are represented as mean ± SD or median (IQR), n=6.

a *p*< 0.05,

b *p*<0.01,

c *p*<0.001, compared with the control group.

d *p*<0.05 compared with the cyclophosphamide group.

The parametric test used was one way ANOVA with Tukey Krammer post hoc test and the Non-parametric test used was the Kruskal Wallis with Dunn’s multiple comparison post hoc test, based on the normality testing.

### Macroscopic and microscopic appearance of testis

**Figure 2:**
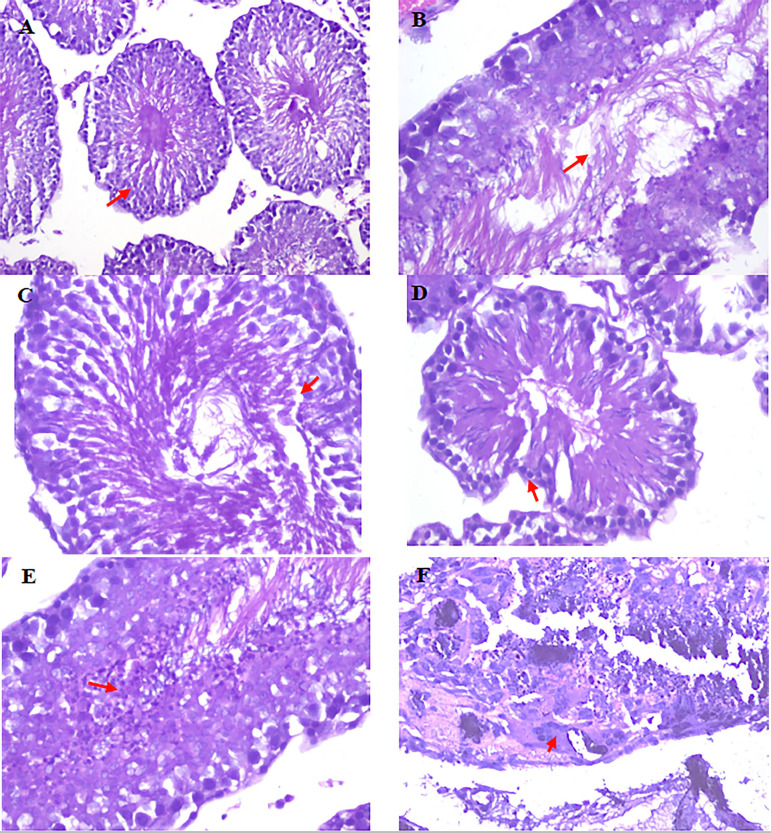
Histopathological changes of rat testis after treatment with *Cleistanthus collinus*. Testis of salinetreated rats showing normal seminiferous tubules and Sertoli cells (A, B). Arrow showing the presence of 3- 4 layers of spermatogenic cells along with mature sperm. The cyclophosphamide-treated group showed reduced spermatogenic layers, with reduced spermatocytes and spermatogonia (C,D). The *Cleistanthus collinus*, 200 mg/kg treated group showed rounded and degenerated sperm cells (E). The *Cleistanthus collinus*, 400 mg/kg treated group showed necrotic seminiferous tubules with the absence of spermatogenesis with granular debris (F). There was also giant cell formation. (H&E stain 200X & 400X).

There was testis calcification in the animals treated with *Cleistanthus collinus* (200 and 400 mg/kg) on gross pathology. Microscopically, there were degeneration of primary spermatocytes and spermatogonia. The layers of spermatogenic cells were reduced in the cyclophosphamide, *Cleistanthus collinus* 200 and 400 mg/kg groups (Figure 2). The control group showed 4-5 layers of spermatogenic cells without any degeneration of sperm cells.

### Effect of *Cleistanthus collinus* on Johnson's scoring and area of the seminiferous tubule

Johnson's testicular scoring in the cyclophosphamide and *Cleistanthus collinus* (200 and 400 mg/kg) groups showed a low score, as given in Table 2. The area of seminiferous tubules was measured and found to be decreased in the cyclophosphamide and *Cleistanthus collinus* (200 and 400 mg/kg) groups is given in Table 2.

**Figure 3:**
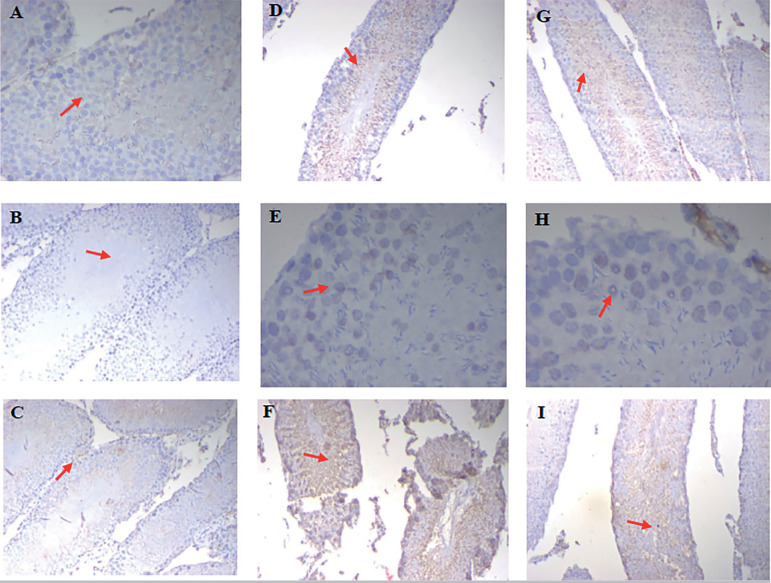
Immunohistochemistry of rat testis for BCL-2, p53 and BAX expression. Saline treated control group animals showing negative stain for BCL-2 and p53 in testis (A & B, 200X.) and BAX positive basal cells and interstitial cells (C, 200X). *Cleistanthus collinus* 200 mg/kg treated animals showing BCL-2 positivity in sperm heads of spermatid and negative in other spermatogenic cells (D), weak p53 positivity (E) and BAX positivity in sperm cells (F). IHC 200X *Cleistanthus collinus* 400 mg/kg group showing BCL-2 positivity in mature sperm and negative in immature sperm cells (G), p53 focal nuclear positivity in spermatocytes (H) and BAX positivity in the spermatozoa(I). IHC 200X.

The control group, which was treated with saline, showed positivity only towards BAX but BCL-2 and p53 were not expressed. The *Cleistanthus collinus* 200 mg/kg group showed Bcl-2 positivity in the spermatid head (Figure 3). The *Cleistanthus collinus* 400 mg/kg group showed BAX positivity in spermatocytes and expression of BCL-2 and p53 in mature sperm cells. In the cyclophosphamide group, BAX was negative and the p53 expression was seen in the stromal cells and endothelial cells. BCL-2 was expressed in the stromal cells (Leydig cells and Sertoli cells).

### Effect of *Cleistanthus collinus* on serum LH, FSH and testosterone

LH (*p*<0.001) and testosterone levels (*p*<0.01) were reduced significantly in the cyclophosphamide-treated group in comparison to the control group (Table 3). In the *Cleistanthus collinus* (400 mg/kg) group, LH, FSH and testosterone levels were reduced significantly (*p*<0.05) in comparison to the control group.

**Table 3.** Effect of *Cleistanthus collinus* on serum LH, FSH and testosterone levels of Wistar rats.

	Control (saline)	Cyclophosphamide 100 mg/kg	*C. collinus* 200 mg/kg	*C. collinus* 400 mg/kg
LH (mIU/L)	13.8 (11.5-19.5)	3.8 (3.5-5.6)^c^	11.95 (9.2-13.7)^e^	8.3 (7.2-10.6)^a^^,^^d^
FSH (mIU/L)	14.68±4.04	13.56±2.40	14.41±1.99	10.30±2.33^a^
Testosterone (nmole/L)	34.0±6.20	19.2±3.90^b^	28.9±7.60	25.70±3.59^a^

Rats received saline or plant extract through oral gavage daily for 28 days. Cyclophosphamide was given through intraperitoneal route once in a week for four weeks. The following day, the animals were slaughtered, and the parameters were assessed. Data are represented as mean ± SD or median (IQR), n=6.

a *p*<0.05,

b *p*<0.01,

c *p*<0.001 compared to the control group;

d *p*<0.05,

e *p*<0.01 compared to the cyclophosphamide group.

The parametric test used was the one-way ANOVA with the Tukey Krammer post hoc test; and the Non-parametric test used  was the Kruskal Wallis with Dunn’s multiple comparison post hoc test, based on the normality testing.

### Effect of *Cleistanthus collinus* on MDA, catalase and GSH

MDA, an indicator of lipid peroxidation was increased in the cyclophosphamide and the *Cleistanthus collinus* 400 mg/kg groups in comparison to the control group (Table 4). Catalase, an antioxidant enzyme, was decreased in the *Cleistanthus collinus* 400 mg/kg group and cyclophosphamide group. But GSH was reduced significantly in all groups compared with the control group.

**Table 4.** Effects of *Cleistanthus collinus* on tissue MDA, catalase and GSH levels of Wistar rats.

	Control (saline)	Cyclophosphamide 100 mg/kg	*C. collinus*200 mg/kg	*C. collinu*s400 mg/kg
MDA nmole/ml	3.24±1.22	7.24±1.10^c^	4.41±2.19^e^	6.78±1.13^b^
Catalase K/ml	2.63±0.83	0.86±0.61^c^	1.68±0.22^d^	1.33±0.45^e^
GSH µg/mg of protein	94.30±7.18	47.53±10.34^c^	62.66 ±1.75^c^^,^^e^	60.71±6.43^c^^,^^d^

Rats received saline or plant extract through oral gavage daily for 28 days. Cyclophosphamide was given through intraperitoneal route once in a week for four weeks. The following day, the animals were slaughtered, and the parameters were assessed. Data are expressed as mean ± SD, n=6.

a *p*<0.05,

b *p*<0.01,

c *p*<0.001 compared with the control group.

d *p*<0.05,

e *p*<0.01 compared with the cyclophosphamide group.

## DISCUSSION

In this study, the effect of different doses of *Cleistanthus collinus* was examined on the reproductive system of male Wistar rats. Our findings, maybe reported for the first time, revealed the reproductive toxicity of the aqueous extract of *Cleistanthus collinus* in male rats on different reproductive, histological and hormonal parameters. Sperm count and motility were lowered in all animals treated with *Cleistanthus collinus.* The morphological abnormalities observed in the *Cleistanthus collinus* treated group suggests the direct damage caused by the plant extract. The reduction seen in the seminiferous tubules was due to the destruction of germ cells and Sertoli cells. The toxic effect of *Cleistanthus collinus* may be mediated through increased production of free radicals, resulting in oxidative stress and resultant damage produced to cells. The expression of apoptotic and antiapoptotic markers was found to be altered, indicating the role of apoptosis on testicular toxicity.

One of the endpoints for assessing the toxicity is body weight, which indicates the presence of toxic substances. Animals treated with *Cleistanthus collinus* (200 and 400 mg/kg) extract showed a decrease in body weight, which was measured after 28 days of its ingestion. Cyclophosphamide also produced a decrease in body weight because of its targeted action on rapidly dividing cells (germ cells) (Kanno *et al.*, 2009). In the control group, there was a gain in body weight, which was a natural process that occurred due to adequate intake of food and water. The findings described above demonstrated the systemic toxicity produced by *Cleistanthus collinus* (Eswarappa *et al.*, 2003).

Reproductive toxicity produced by any substance may cause a change in the reproductive organ weight. Hence testis and epididymal weight were studied to evaluate reproductive toxicity. *Cleistanthus collinus* treatment (200 and 400 mg/kg) reduced the testicular and epididymal weights when compared with the control group. Parasuraman *et al*. (2014) studied the sub-chronic toxicity caused by the individual compound of *Cleistanthus collinus* i.e, cleistanthin A and B on different organ systems (Parasuraman *et al.*, 2014). In their study cleistanthin A showed a gain in testicular weight, whereas cleistanthin B did not cause any change in testicular weight. Animals treated with cyclophosphamide showed a reduction in testicular and epididymal weights due to its targeted action on rapidly dividing cells leading to the loss of spermatogenic cells (Elangovan *et al.*, 2006). A similar effect might have also been produced by *Cleistanthus collinus,* leading to a decrease in testis weight. In addition, the reduction in hormone levels, especially testosterone, should directly impact the weight of the testis. In the case of the epididymis, there was certainly a decrease in weight (especially the 400 mg/kg dose), though not statistically significant. The reproductive organ weight may also be influenced by the inflammatory changes that occur in these organs due to the toxin, resulting in fluid buildup. So, the changes in organ weight must be interpreted by considering different factors (Azenabor *et al.*, 2015).

Sperm count, motility and morphology are the most important parameters in the assessment of spermatogenesis quality. In our study, sperm count was reduced in animals treated with 400 mg/kg; whereas in the 200 mg/kg group a decrease in sperm count did not occur. This showed dose-related reproductive toxicity of the aqueous extract of *Cleistanthus collinus*. The cyclophosphamide-treated animals also showed reduced sperm count compared with the control group. Sperm motility was affected in animals treated with 400 mg/kg of *Cleistanthus collinus,* when compared with the control group. Sperm motility is strictly regulated by the tail end of it. Energy is needed for the forward movement of sperm and this energy (ATP-adenosine triphosphate) is mainly produced from mitochondria, which are present in the middle piece of the sperm (Piomboni *et al.*, 2012; Ruiz-Pesini *et al.*, 1998). In our study sperm motility was affected by *Cleistanthus collinus,* which may be due to the mitochondrial damage caused by the plant extract. The plant extract with the poisonous property was responsible for the damage in the cell organelle (mitochondria) and resulted in decreased ATP synthesis needed for sperm motility. Cyclophosphamide is a cytotoxic drug that caused cell damage, resulting in reduced sperm motility among the animals treated with it. The sperm morphological examination revealed abnormalities in all treatment groups, but not in the control group. The morphological alteration produced by *Cleistanthus collinus* was more in the 400 mg group than the 200 mg group and was comparable with the cyclophosphamide group. These changes may be attributed to the imbalance between the oxidant and antioxidant levels. This was supported by a decrease in antioxidant levels (catalase and GSH), which was demonstrated in our study.

The hormones involved in the regulation of spermatogenesis are LH, FSH and testosterone. Reproductive toxic drugs may interfere with them. The positive control drug and the well-known reproductive toxicant agent, cyclophosphamide-treated animals showed decreased levels of LH and testosterone without affecting the FSH level. *Cleistanthus collinus* at higher doses (400 mg/kg) showed a reduction in the levels of LH, FSH and testosterone. This demonstrated the reproductive toxic nature of the plant extract affecting the endocrine system (Tuchinda *et al*., 2006). LH and FSH play a vital role in steroidogenesis, where LH causes the Leydig cells to synthesize testosterone; whereas FSH acts on Sertoli cells to produce androgen-binding protein. Numerous herbal drugs can target these Leydig cells, hinder spermatogenesis and produce infertility (D'Cruz *et al.*, 2010). Reduction in the levels of testosterone, LH and follicle-stimulating hormone was reported when the crude methanol extract of *Quassia amara* was administered to male albino rats. Administration of the methanol extract of *Sarcostemma acidum* to male albino rats for 60 days was shown to cause a decrease in the number of mature Leydig cells, and an increase in the degeneration of the Leydig cell population. Suppression of the activities of steroidogenic enzymes including the P450 side-chain cleavage enzyme, 3 β-hydroxysteroid dehydrogenase, 17 α-hydroxylase, 20 α-hydroxylase and 17 α-hydroxysteroid dehydrogenase, was observed when primary mouse Leydig cells were incubated with varying concentrations of crude *Toona sinensis* (D'Cruz *et al.*, 2010). *Carica papaya* seed extracts were reported to produce pronounced hypertrophy of pituitary gonadotrophs and degeneration of Leydig cells, in sexually mature Wistar rats. Similarly, *Cleistanthus collinus* may decrease the number of mature Leydig cells or suppress the activities of steroidogenic enzymes. The alteration in hormonal levels might have suppressed spermatogenesis, which is regulated by the hypothalamic-pituitary-gonadal axis (Ghosh *et al.*, 2002).

Antioxidants are the enzymatic or non-enzymatic substances involved in fighting against the free radicals which cause cell injury. In our study, we assessed the levels of two antioxidants, catalase (enzymatic antioxidant) and GSH (non-enzymatic antioxidant), which are needed to protect the cells against free radicals. Catalase and GSH were reduced in the testicular tissue homogenate of *Cleistanthus collinus* 400 mg/kg and cyclophosphamide groups compared with the control group. This may be due to the increased levels of free radicals, which have consumed the antioxidants and resulted in low levels of it. This reveals the toxic nature of *Cleistanthus collinus* extract on testicular tissue. Malondialdehyde (MDA) is another marker for assessing cellular injury whose levels are increased due to the lipid peroxidation caused by free radicals. MDA was increased in the *Cleistanthus collinus* 400 mg/kg group and the cyclophosphamide group, but not in the *Cleistanthus collinus* 200 mg/kg group. It showed that the plant extract increased cellular stress due to increased free radical synthesis, ultimately leading to the rise of MDA. These findings indicate that *Cleistanthus collinus* has the property of causing cell injury by generating free radicals. Jayanthi *et al*. (2009) studied the effects of melatonin on the oxidative damage caused by *Cleistanthus collinus* plant extract (Jayanthi *et al*., 2009). In their study, an aqueous extract of *Cleistanthus collinus* caused damage to the brain tissue where the levels of GSH and catalase were reduced, which supported our study findings (Parasuraman *et al*., 2009; Ghobadi *et al*., 2017).

Morphometric analysis deals with the measurement of cells of interest in terms of diameter, area and perimeter (Narayana, 2008). In this study, the area of the seminiferous tubules was reduced significantly in all treatment groups when compared to the control group. The area of seminiferous tubule is dependent on the spermatogenic cells. From the above finding, it was evident that the aqueous extract of *Cleistanthus collinus* had exerted its toxic activity on rapidly dividing cells and thereby reduced the area of the seminiferous tubule.

Histopathology of the testis showed degeneration and necrosis of sperm cells in animals treated with 200 and 400 mg/kg of *Cleistanthus collinus*. This explained the direct damage caused by *Cleistanthus collinus* on the rapidly dividing cells of testes. Similar findings were observed in the cyclophosphamide group. Johnson's scoring is a quantitative assessment of the seminiferous tubules. A score of 1-10 was given based on the presence of spermatogenic cells and Sertoli cells. A high score indicates the normal function of the testes, whereas a score will be low in the presence of necrosis, calcification and inflammation of germ cells and Sertoli cells of the testicular tissue (Johnsen, 1970). With a 200 and 400 mg/kg dose of *Cleistanthus collinus* group, there was degeneration and necrosis of sperm cells. The cyclophosphamide group demonstrated reduced spermatogenic layers and primary spermatocytes. From the results of testicular scoring, it was evident that the animals treated with *Cleistanthus collinus* extract damaged the testicular tissue.

Like histopathology, the immunohistochemical analysis also provides direct evidence when compared to other biochemical investigations. Normally BAX (apoptotic factor) is expressed in the testicular tissue to remove the immature and incompetent sperm cells by programmed cell death, causing a proper control over the spermatogenesis process (Ghobadi *et al.*, 2017). Other apoptotic (p53) and anti-apoptotic factors (BCL2) are not expressed normally (Dündar *et al*., 2005; Beumer *et al*., 1998).

As expected, the control group expressed positivity for BAX (apoptotic factor)m which is needed for the normal development of testes and spermatogenesis. P53 and BCL-2 were not expressed in the control group. This happened because of an adequate balance between the oxidants and antioxidants. The *Cleistanthus collinus* 200 mg/kg group showed BCL-2 positivity in the spermatid head. Expression of BAX was increased compared to the control group. This clearly showed the change in the expression of normal regulatory apoptotic factors. Apoptosis was increased in the testicular tissue due to irreparable damage caused by the excess reactive oxygen species (Oldereid *et al.*, 2001; Ozaki & Nakagawara, 2011).

The *Cleistanthus collinus* 400 mg/kg group showed BAX positivity in spermatocytes. BCL-2 and p53 were expressed in mature sperm cells. Expression of both apoptotic and anti-apoptotic factors in mature sperm cells directly tell us that these cells were the target of free radical injury. Increased BAX expression implied increased apoptosis in spermatocytes. In the cyclophosphamide group, BAX was negative and p53 expressed positivity in stromal cells and endothelial cells. Similarly, BCL-2 was also expressed in stromal cells. Leydig cells and Sertoli cells are the stromal cells involved in testosterone synthesis and provide nourishment, respectively. Cyclophosphamide had caused damage to these stromal cells and resulted in the expression of apoptotic (p53) and antiapoptotic factors (BCL-2). Alteration in the apoptotic factors demonstrated the cell damage caused by the cyclophosphamide drug (Dündar *et al.*, 2005; Beumer *et al.*, 1998).

This is the first study conducted to assess the male reproductive toxicity of *Cleistanthus collinus*. This information must be kept in mind while developing compounds from *Cleistanthus collinus,* since it contains phytochemicals with pharmacological properties which can act as drugs. We did not characterize this extract in the present study. Therefore, it is difficult to know which compounds are present in the plant that could have caused the reproductive impact. Further studies should be carried out for knowing the reproductive toxic effect on long term exposure to this plant, along with developmental toxicity. The reversibility of infertility in *Cleistanthus collinus* extract was not assessed. *Cleistanthus collinus* contains many chemicals more notably cleistanthin A, cleistanthin B, diphyllin and collinusin. Such active phytochemicals isolated from C*leistanthus collinus* should be studied for male and female reproductive toxicity.

## CONCLUSION

Aqueous extract of *Cleistanthus collinus,* when administered to male Wistar rats, produced reproductive toxicity. This was reflected as a reduction in sperm count and motility in male Wistar rats. The morphology of spermatozoa was also affected by the ingestion of the plant extract. The levels of luteinizing hormone, FSH and testosterone were reduced after exposure to *Cleistanthus collinus* extract. The levels of enzymatic antioxidant, catalase and non-enzymatic antioxidant GSH were reduced in animals treated with *Cleistanthus collinus* extract, indicating an imbalance between the pro-oxidant and antioxidant systems. There was a rise in the MDA levels in animals treated with *Cleistanthus collinus*, demonstrating the cell membrane damage, which was due to lipid peroxidation. There was the destruction of primary spermatocytes, spermatids and reduced thickness of the basal layer. The area of seminiferous tubule was also reduced in animals treated with *Cleistanthus collinus* extract, indicating loss of the germ cells needed for sperm production. BCL2 (anti-apoptotic factor) and p53 (apoptotic factor) were expressed in animals treated with *Cleistanthus collinus,* indicating their role in the toxic effect. All the above findings suggested the reproductive toxic nature of Cleistanthus collinus extract on male Wistar rats.
